# Atomic picture of elastic deformation in a metallic glass

**DOI:** 10.1038/srep09184

**Published:** 2015-03-17

**Authors:** X. D. Wang, S. Aryal, C. Zhong, W. Y. Ching, H. W. Sheng, H. Zhang, D. X. Zhang, Q. P. Cao, J. Z. Jiang

**Affiliations:** 1 International Center for New-Structured Materials (ICNSM), Laboratory of New-Structured Materials, State Key Laboratory of Silicon Materials, and Department of Materials Science and Engineering, Zhejiang University, Hangzhou, 310027, People's Republic of China; 2 Department of Physics and Astronomy, University of Missouri-Kansas City, Kansas City, MO 64110 USA; 3 School of Physics, Astronomy and Computational Sciences, George Mason University, Fairfax, VA22030, USA; 4 Department of Chemical and Materials Engineering, University of Alberta, Edmonton, Alberta, T6G 2V4, Canada; 5 State Key Laboratory of Modern Optical Instrumentation, Zhejiang University, Hangzhou, 310027, People's Republic of China

## Abstract

The tensile behavior of a Ni_60_Nb_40_ metallic glass (MG) has been studied by using *ab initio* density functional theory (DFT) calculation with a large cell containing 1024 atoms (614 Ni and 410 Nb). We provide insight into how a super elastic limit can be achieved in a MG. Spatially inhomogeneous responses of single atoms and also major polyhedra are found to change greatly with increasing external stress when the strain is over 2%, causing the intrinsically viscoelastic behavior. We uncover the origin of the observed super elastic strain limit under tension (including linear and viscoelastic strains) in small-sized MG samples, mainly caused by inhomogeneous distribution of excess volumes in the form of newly formed subatomic cavities.

In general, the relationship between the stress and strain of materials follows the classical Hooke's law when the stress is in the elastic regime^1^. After the stress is released, the strain recovers to zero linearly without any hysteresis. The stress-strain relationship may deviate from the linear mode when creep^2^, stress-induced phase transition^3^ or yielding^4^ etc., happens. Metallic glasses (MGs), a new class of engineering materials, have nearly 2% elastic strain limit, which is much higher than that of their crystalline counterparts. Moreover, it has been shown in recent experiments that this value can be even higher, for instance, about 3%, 4.4%, and 6.6% respectively for nanometer-sized MG samples cut from a bulk Zr-Ti-Co-Be MG^5^, a ribbon Cu-Zr MG^6^, and a 50 nm-film Ni-Nb MG^7^. Although much work has been done on elasticity of traditional glassy materials^8^,^9^,^10^, the above results still triggered much interest in the elastic behavior of MGs. The local heterogeneity in elastic properties within the nanoscale was observed in bulk MG samples^11^. It was also speculated that MGs at room temperature may consist of tightly bonded atomic clusters and loosely bonded free volume regions^12^. During the elastic deformation, the tightly bonded regions were assumed to undergo pure elastic response whereas the loosely bonded regions to behave like supercooled liquids, thus showing a viscoelastic feature on the whole. Using x-ray diffraction and bond orientational anisotropy analysis, Dmowski *et al.* demonstrated that 3/4 volume fraction of MGs deforms elastically and the rest one quarter belongs to the inelastic portion^13^. More recently, Ding *et al.* proposed a solidity/liquidity index, based on bond orientational anisotropy to assess the varying degree of local structures in MGs upon shear stress and the heterogeneous elasticity^14^,^15^ by classic molecular dynamics (MD). However, the atomistic mechanism underlying the superior elastic properties of MGs is still not fully understood. How to separate the solid-like regions from the liquid-like regions on the atomic scale? What difference in elastic responses between them? What is the major factor behind to control these? Such questions still remain and thus it is imperative to investigate how the glassy structures respond to the stress on the atomic level in MGs in the elastic regime. In this paper, we apply the *ab initio* density functional theory(DFT) to calculate a Ni_60_Nb_40_ MG model containing 1024 atoms (an extremely large number for DFT calculations) upon tension and trace the structural evolution of atoms with increasing tensile strain from 0 to 7%. It is found that not only single atoms but also major polyhedra have inhomogeneous responses to external tensile stress when the strain is over 2%. These heterogeneities suggest that the elasticity of MGs becomes viscoelastic by forming new cavities that evolve with external tensile stress, resulting in the observed super elastic strain limit in small-sized MG samples.

Fig. 1a illustrates the comparison in structure factor *S(q)* and pair distribution function *G(r)* between the simulation data calculated from the fully relaxed configuration under zero stress and the experimental data extracted from high energy x-ray diffraction (100 keV) on the BW5, Hamburg, using a ribbon sample prepared by melting spinning. More experimental details are given in Supplementary Materials (SM). Due to time scale restriction, the simulation-produced sample cannot reach the atomic relaxation level in the ribbon sample. However, the general structure features of the real-world MG can be captured (e.g., peak position and shape), suggesting that the simulation data could be still reliable. Fig. 1b shows the stress vs. strain curve of Ni-Nb MG under loading and unloading processes. In the loading process, when the macro-strain is less than 2%, the stress increases almost linearly with the strain. However, when the strain is further increased to 7%, the stress-strain relationship deviates from the linear mode, exhibiting an obvious nonlinearity in the strain range from 2 to 7%. Upon unloading, the stress vs. strain curve does not follow the loading curve but exhibits a linear relationship, indicating that about 6% of the elastic part can be recovered and only nearly 1% of the inelastic or/and plastic portion still remains within the present simulation timescale after complete stress release, which is close to our experimental value 6.6%^7^.

We calculate the atomistic local strain by comparing the changes in atomic positions between the present and a reference state. The local shear strain invariant *η*^Mises^ of each atom in the sample is calculated using equation^16^: 
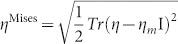
, where *η* and *η_m_* are local Lagrangian strain and local hydrostatic strain for that atom, respectively. To calculate the 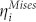 of each atom, we use a reference configuration that has 1% strain lower. The values of *η*^Mises^ can reveal whether the local shear strain distribution is homogeneous in the elastic regime for all atoms. Fig. 2a shows the distribution of von Mises strain for the total atoms with increasing tensile strain. When the tensile strain increases to 2%, the distribution of von Mises strain for all atoms remains localized, indicating that most atoms have the same local shear strain around 0.008. It is fully consistent with the 2% elastic strain limit for MG bulk samples. After that, the distribution of von Mises strain becomes delocalized, spanning a wide range from 0.002 to 0.015 when the tensile strain reaches about 7%. These results point the fact that the atoms in the sample have significantly diverse responses to the external tensile stress when the tensile strain is larger than 2% and the sample mainly undergoes viscoelastic deformation.

It is commonly believed that metallic glasses consist of different types of polyhedra, which have a wide distribution^17^. In the dense random hard sphere model, the hard spheres closely pack together and form solid-like regions. Naturally, the volumes with disconnected cavities make up the void regions. The deformation behavior of MGs was demonstrated to have close relations with their inside free volume or open volume changes^18^,^19^. By using the algorithm proposed by Sastry^20^,^21^ (more details seen in SM and Fig. S1), the open volume regions in Ni_60_Nb_40_ alloy can be evaluated by setting suitable exclusion radii (about 1.39 times Schmidt radii) for Ni and Nb atoms, respectively. In doing so, only those relatively large cavities are enumerated and the tiny ones, e.g. tetrahedral and octahedral cavities, are excluded. Thus, in the so-called “solid” regions, no cavities are involved. By this means, the atoms can be grouped as locating in the “solid” and cavity regions. As shown in Fig. 2b, with increasing tensile strain, the distribution of von Mises strain of atoms either in the “solid” or cavity regions becomes broader, indicating the increased local heterogeneities with external strains. However, the main peak of the von Mises strain for the atoms in the cavity regions moves to the large strain values, while for those in the “solid” regions shifts to the low ones. This result demonstrates that most atoms are involved in the inelastic deformation when the strain is larger than 2%, whereas the atoms in the cavity regions have slightly larger local strain than those in the “solid” regions. This is consistent with local atomic displacements of atoms changing with tensile strain in both regions, as shown in Fig. S2.

Figs. 3a–c demonstrate that the total number of atoms near the cavities increases with increasing tensile strain, especially for the strain from 3% to 7%. The fraction of atoms near the cavities is estimated to be about 32.7% at zero strain, about 35.1% at 3% strain and then rapidly increases to 41.0% at 7% strain. Compared to 25% atoms undergoing inelastic deformation estimated in Ref. 13, our results demonstrate that this value could be even larger in the small-sized MG samples. It is also found that the tensile stress applied favors the formation of new voids after 3% strain. The larger the tensile strain, the more voids are formed in response. During deformation, the formation of new voids is a dynamical process associated with the annihilation of existing ones, and is found to be randomly but inhomogeneously distributed. Some of them are inclined to form near the existing ones and accumulate to form relatively big cavities when the strain is larger than 3%. Fig. 4a shows the total volume change of the simulation cell and Fig. 4b exhibits the cavity volume changes during loading and unloading. Compared with the stress vs. strain curves in Fig. 1b, the total volume change exhibits almost the similar variation trend. However, some difference still exists. The total volume change deviating from the linear mode is not so significant when the strain is from 2 to 4%. Upon unloading, the total volume can be nearly recovered while the rest strain still remains about 1.6%. In contrast, the cavity volume change is more consistent with the stress-strain curves in Fig. 1b. During the loading process, the cavity volume increases linearly in the pure elastic strain range. It increases much faster than predicted by the linear relationship and can be fitted by an exponential function when the deformation is in the inelastic regime (2% to 7% strain). Due to the creation of more cavities, viscoelasticity becomes dominant. Upon unloading, cavity volume and stress (in Fig. 1b) almost linearly decrease with strain, but the residual cavity volume in the sample is always larger than the value at the same strain during loading. Fig. 4c exhibits a trend that the cavity volume increase results from both the increases of cavity number and size with increasing tensile strain. Our results obtained here indicate that the viscoelasticity of MGs under tension is mainly governed by fast proliferation and slow annihilation of excess volumes in the form of subatomic cavities at high stresses.

To determine the response of local atomic environment to stress, the Voronoi tessellation method^22^ is adopted with a cutoff of 3.6 Å, corresponding to the first minimum in pair correlation function (PCF) curves. At the same time, different types of atomic clusters are traced to see how they deform upon tension. Figs. 5a–b illustrate the fraction changes of Voronoi polyhedra centered by Ni in both “solid” and cavity regions with increasing strain. The evolution of polyhedra centered by Nb atoms can be seen in Fig. S3. It is found that the fractions of major polyhedra in the “solid” regions on the whole do not change so drastically as opposed to those near the cavities. The top three types of polyhedra centered by Ni atoms in the “solid” regions are successively <0,2,8,2>, <0,2,8,1> and <0,0,12,0>, as shown in Fig. 5a, each of which has a population larger than 10%. With increasing tensile strain, the fraction of <0,2,8,2> in the solid regions tends to decrease while the fraction of <0,0,12,0> remains almost unchanged. These can also be reflected from the large distortions for <0,2,8,2> polyhedra and less distortions of perfect <0,0,12,0> icosahedra induced by stress, as shown in Fig. 5c, consistent with the reports in Refs. 23,24. Interestingly, the fractions of <0,2,8,1> and <0,3,6,4> centered by Ni atoms near the cavity region in Fig. 5b significantly increase, while those of <0,2,8,2> and <0,0,12,0> are reduced accordingly in comparison to the “solid” regions (see Fig. 5a). These results demonstrate a structural heterogeneity in atomic structures in MG from “solid” to cavity regions. An opposite evolution trend is found in the fractions of <0,2,8,2> and <0,3,6,4> polyhedra, as reflected from the trajectory of atoms analysis in Fig. 5d, in which two cluster types <0,2,8,1> and <0,3,6,4> coexist at zero strain. At 6% stain, the structure of polyhedron <0,2,8,1> does not change much, while the <0,3,6,4> evolves into the <0,2,8,2> via the expansion and breaking of A–B bonds, and simultaneous contraction and formation of C–D bonds. As expected, bond orientational anisotropy indeed exists in MGs when directly tensioned in the elastic regime as previously suggested by Egami *et al.*^25^. However, as shown in Fig. S4, the value of bond orientational parameter 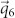
26 keeps almost the same at various strain levels, which may be explained by the high rigidity of icosahedron-like clusters. In contrast, a slight shift in 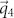 parameter to big values implies the existence of preferred bond orientations during tension, e.g., cubic symmetry, although the variation amplitude is not so abrupt in the studied elastic regime. Thus, the viscoelastic behavior of metallic glasses above 2% strain is mainly controlled by the generation, evolution and annihilation of stress-induced defects, e.g., subatomic cavities.

It is found that the elastic strain limit of a Cu_50_Zr_50_ MG in compression is smaller than that in tension as shown in Fig. S5a. When the stress is over 0.8 GPa, the viscoelasticity becomes easy to start under tension. Fig. S5b–c indicates that the compression decreases the total volume and also subatomic cavities, thus reducing the viscoelasticity accordingly compared to the tension. In Refs. 27 the viscoelasticity of MGs was supposed to correlate with the amount of irreversible shear transformation zones (STZs). However, the irreversible STZs most likely are the sources of viscoplastic deformation^28^ rather than viscoelasticity of MGs. In contrast, our results demonstrate that the subatomic cavity change could give a general picture for describing the elasticity of MGs.

Compared to their crystalline counterparts, MGs may exhibit high yield strength approaching the theoretical limits (e.g., ~*E*/10)^29^ due to the lack of dislocations in the material. In bulk MG samples, except the excess free volume, the probability for the occurrence of casting defects increases, which may act as stress concentration sources^30^. With increasing tensile stress (beyond the experimentally observed yield strength), the material usually fails in a catastrophic fashion along a major shear band. This most likely occurs at the casting defects, corresponding to a strain value usually less than 2%. This value is consistent with the linear elastic strain limit obtained in our simulations. By contrast, in nanometer-sized MG samples the chance having casting defects becomes slim. Most of the newly-formed cavities during tension are rather randomly distributed, which restrain the formation of major shear band by dispersing locally concentrated stresses, and thus lead the deformation into the viscoelastic regime. Consequently, nanometer-sized MGs can exhibit higher values in elastic strain limit, as observed in Refs. 5,6,7.

In summary, the tensile behavior of a Ni_60_Nb_40_ MG has been investigated by using *ab initio* DFT simulation with a large supercell containing 1024 atoms. With increasing tensile strain, the local atomic strain becomes more heterogeneous. The major polyhedra near the cavities show larger variations in fractions than those in the solid-like regions. These heterogeneities suggest that the elasticity of MGs is intrinsically viscoelastic when the strain is over 2%. New cavities as well as bond orientational anisotropy are detected, resulting in the observed super elastic strain limit. This study uncovers the origin of the observed super elastic strain limit (including linear and viscoelastic strains) in small-sized MG samples, mainly caused by inhomogeneous distribution of excess volumes in the form of newly formed subatomic cavities, and will shed new light on the understanding of the deformation behavior of MGs.

## Methods

Numerical simulation, e.g., molecular dynamics (MD), is a powerful tool that can track the trajectory of atoms in MGs upon deformation^19^,^31^,^32^. However, it is often restricted by the availability of precise potentials in the classic MD (see Supplementary Fig. S5) or by the limited atom numbers in the *ab initio* MD. In principle, DFT is a parameter-free accurate method that has been demonstrated to yield comparable results with the experiments.

We build a cubic box containing 1024 atoms (614 Ni and 410 Nb) to simulate the heating and cooling processes by LAMMPS^33^ using an embedded-atom-method (EAM) potential of Ni-Nb with periodic boundary conditions in three directions. The sample is heated to 2400 K, and then quenched to 300 K at a cooling rate of 10^11^ K/s with NPT ensemble. The obtained configuration is further fully relaxed using Vienna *ab initio* Simulation Package (VASP)^34^ with periodic boundary conditions but without any constrictions in cell shape and atomic positions. The box size obtained is a = 24.362 Å, b = 24.341 Å, c = 24.096 Å, *α* = 89.53°, *β* = 89.87° and *γ* = 90.03° with the final density of about 8.571 g/cm^3^, in good agreement with the experimental data^7^ of about 8.5 ± 0.05 g/cm^3^. The elastic coefficients and mechanical properties of this fully relaxed model are calculated using a strain vs. stress approach^35^ to obtain the bulk mechanical properties (bulk modulus *K* = 192.7 GPa, shear modulus *G* = 43.6 GPa, Young's modulus *E* = 121.7 GPa, averaged Poisson's *ν* = 0.395) including directionally dependent Poisson's ratios based on the Voigt-Reuss-Hill (VRH) approximation, which are comparable with the experimental data of *K* = 174.9 GPa, *G* = 48.2 GPa, *E* = 132.0 GPa and *ν* = 0.37 reported for a Ni_50_Nb_50_ MG^36^. The use of directionally dependent Poisson's ratio in uniaxial tensile simulation is crucial. The model is then subjected to successive extension in the x direction with an interval of 1% elongation. At each step, the model is fully relaxed using VASP and the cell dimensions in the other directions and the volume is adjusted according to the directionally dependent Poisson's ratios. The stress and the atomic coordinates of the model at each step are then recorded. The total time used on this project is over 2 million processor hours on Hopper machine at National Energy Research Scientific Computing Center (NERSC).

## Author Contributions

J.Z.J. designed the research. X.D.W., S.A. and W.Y.C. initiated the research and conducted the simulations. X.D.W., H.W.S., C.Z., H.Z., D.X.Z., Q.P.C., W.Y.C. and J.Z.J. analyzed the data. X.D.W., H.W.S. and J.Z.J. wrote the manuscript.

## Supplementary Material

Supplementary Information Supplementary materials

## Figures and Tables

**Figure 1:**
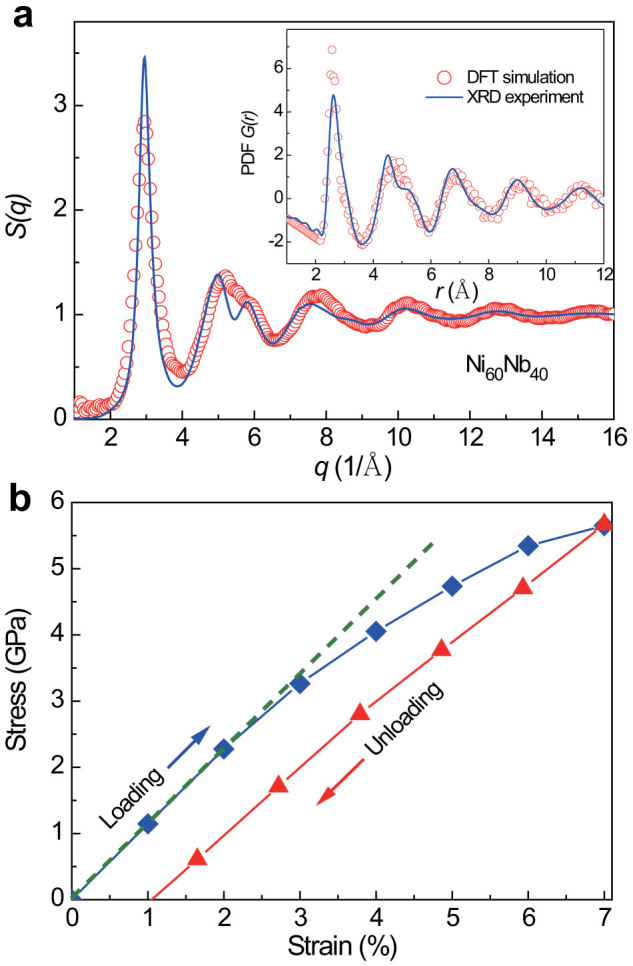
(a) Comparison in *S(q)* and *G(r)* (inset) extracted from XRD measurement (solid line) and VASP simulation (open circle) for the Ni_60_Nb_40_ MG, respectively, showing good agreement between them.(b) Stress-strain curve for a Ni_60_Nb_40_ MG upon loading and unloading produced by *ab initio* DFT simulations.

**Figure 2:**
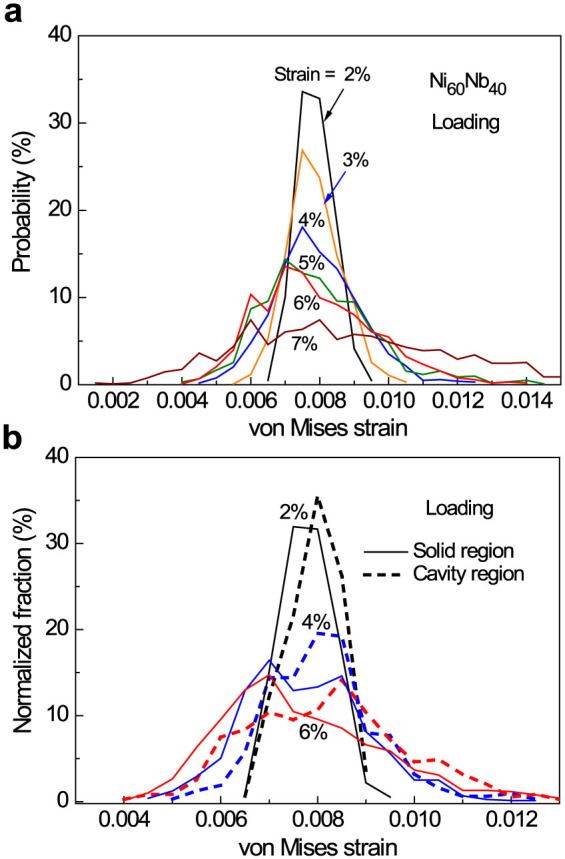
(a) Distribution of von Mises strain for the atoms changing with tensile strain, showing that the larger the tensile strain, the more heterogeneous distribution of the local atomic strain.(b) von Mises strain distributions for the atoms in both the solid regions and the cavity regions changing with tensile strain, respectively.

**Figure 3:**
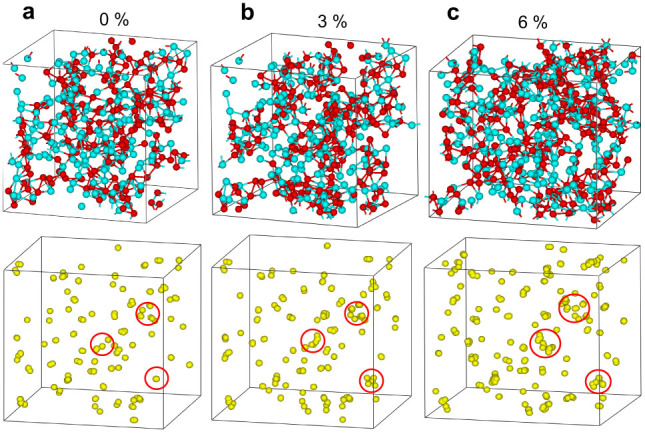
The atoms near the cavities and cavities themselves increases with tensile strain of (a) 0%, (b) 3% and (c) 6%, respectively (Red: Ni, Cyan: Nb and Yellow: cavities). Only cavity center positions are plotted without concerning their shapes. The new cavities are randomly formed but tend to increase their numbers in some localized regions with increasing external tensile strain.

**Figure 4:**
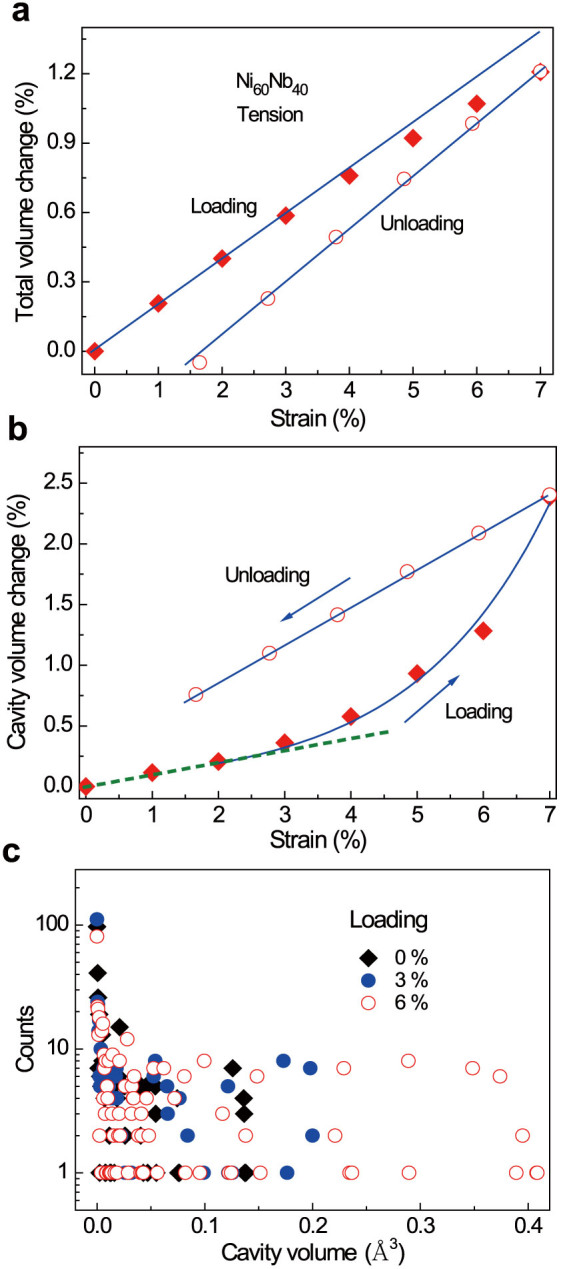
(a) Total volume change and (b) Cavity volume change dependent with tensile strain (Solid squares for loading and open circles for unloading).The solid lines show the cavity volume change almost exponentially increases upon loading and linearly recovers during unloading when the strain is over 2%, implying that the atomic level cavitation may take place upon loading at high strains (c) Not only cavity numbers but also sizes increasing with tensile strain of 0%, 3% and 6% upon loading.

**Figure 5:**
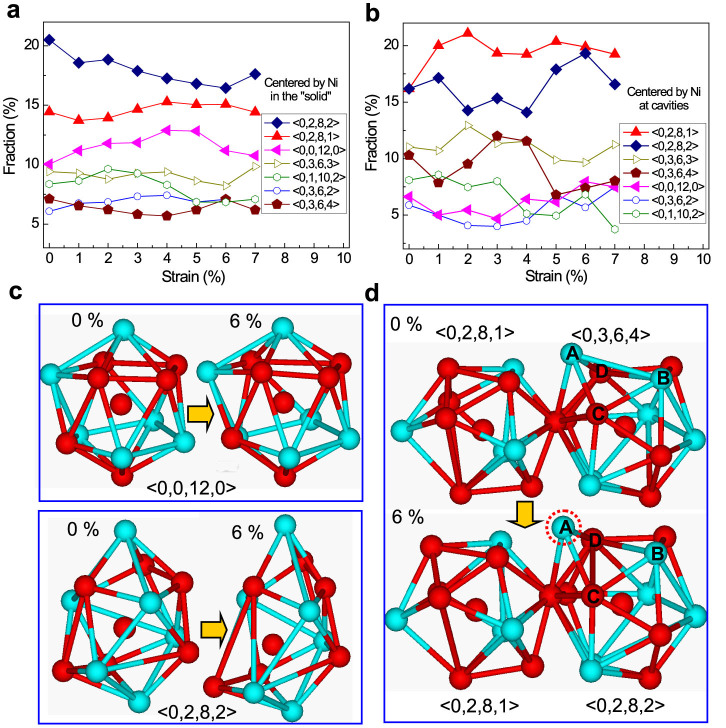
Fractions of major Voronoi polyhedra centered by Ni atoms (a) in the “solid” regions and (b) near the cavities change with tensile strain. (c) Structure changes of icosahedron <0,0,12,0> and polyhedron <0,2,8,2>. (d) Two clusters of <0,2,8,1> and <0,3,6,4> under zero strain and strain of 6%, in which bond orientational anisotropy is observed in the elastic regime for Ni_60_Nb_40_ MG.
