# Supplementary material for: Atomic picture of elastic deformation in a metallic glass
Source: Sci Rep. 2015 Mar 17;5:9184. doi: 10.1038/srep09184 (PMC4361865; doi:10.1038/srep09184)
Supplement: Supplementary Information — Supplementary materials [file srep09184-s1.pdf]

Supplementary materials

Atomic picture of elastic deformation in a metallic glass

X.D. Wang,[a] S. Aryal, C. Zhong, W.Y. Ching,[b] H.W. Sheng, H. Zhang, D.X. Zhang, Q.P. Cao, J.Z. Jiang[c]

Author to whom correspondence should be addressed: (a) wangxd@zju.edu.cn, (b) ChingW@umkc.edu and (c) jiangjz@zju.edu.cn

This PDF file contains:

Supplementary text

Fig. S1, Fig. S2, Fig. S3, Fig. S4, Fig. S5 and Fig. S6

Full reference list

1. Model construction

A pre-built cubic model containing 1024 atoms (614 Ni and 410 Nb) with periodic boundary conditions in three directions is fully relaxed using Vienna *ab initio* Simulation Package (VASP) with a canonical NVT (constant atom number, volume, and temperature) ensemble [1]. A projected augmented wave method [2] and generalized gradient approximation [3] and PAW-PBE potential [4] were used to describe electron-ion interactions. We used a cutoff energy of 500 eV, a relatively high accuracy for the ground state electronic convergence criterion (10^{-5}eV) and force convergence limit (10^{-2}eV/Å). The stress level of the final equilibrium structure is less than 0.1 GPa. The relaxation of the present model

imposes no restrictions on the volume and lattice vectors of the periodic supercell. Since our model is sufficiently large, only one *K*-point at $\Gamma(0,0,0)$ is used.. Pair correlation function *g(r)*, which describes the probability of finding an atom at a distance *r* from a reference atom, is related to the average number of atoms found within a given volume of shell. For isotropic system *g(r)* can be averaged over angles and estimated from atomic configurations by calculating an average number of atoms at distances $r \sim r + \Delta r$ from any given atom. To define the probability to find an atom at a distance *r* from a given atom, *g(r)* can be calculated up the distance r_{max} that should not be longer than the half of the size of the cell using the equation: $g(r) = N/(4\pi r^2 \Delta r) = \frac{1}{4\pi N r^2 \rho_0} \sum_{j=1}^{N} \sum_{\substack{i=1 \\ i \neq j}} \delta(r - r_{ij})$. Then pair distribution function can be defined by $G(r) = 4\pi r \rho_0 (g(r) - 1)$. And the structure factor, which reflects the amplitude of the wave scattered by the sample by summing the amplitude of scattering from each atom in the configuration, can be alternatively calculated by integration over pair correlation function, $S(q) = 1 + \int_0^\infty 4\pi r^2 \rho_0 g(r) \frac{\sin(qr)}{qr} dr$. In experiments, a $Ni_{60}Nb_{40}$ metallic glass (MG) ribbon sample was prepared by melt spinning under purified argon atmosphere. The slices of the sample were transferred into a capillary with diameter of 2 mm. The diffraction patterns were measured by using high energy x-ray diffraction (HEXRD) at the BW5 station (energy ~100 keV) of HASYLAB, Hamburg. The total scattering intensity *I(q)* (versus scattering vector $q = 4\pi \sin\theta / \lambda$) was extracted using the software package FIT2D[5]. The structure factor *S(q)* and the pair distribution function (PDF) *G(r)* could be obtained by the program of PDFgetX2 [6]. As the agreement in *S(q)* and *G(r)* between the simulation data and the experimental data can be achieved, it suggests that the atomic configuration reproduced by

simulation method is reasonable and could be possibly used for atomic structure analysis for this MG.

2. Cavity volume calculation

Geometry of metallic glasses can be regarded as spheres randomly packing in three dimensional spaces. On the large scale, they seem uniform and isotropic. However, on the atomic scale, they are really anisotropic and can be divided into the occupied regions and void regions (regions not occupied by the spheres). In general, such void space is composed of disconnected regions or cavities, which plays an important role in the properties of metallic glasses, leading to the famous free volume model of MGs[7]. For the hard sphere system, Sastry *et al.* [7] proposed a method that can precisely calculate the volume and surface areas associated with such cavities. The first step in this algorithm is to generate the corresponding Voronoi and Delaunay tessellations. Both methods divide the space into non-overlapping regions and are dual to each other as shown in Fig. 1 in ref. [8]. If a set of Voronoi vertices and edges belong to a cavity or cluster, the union of Delaunay simplices corresponding to the

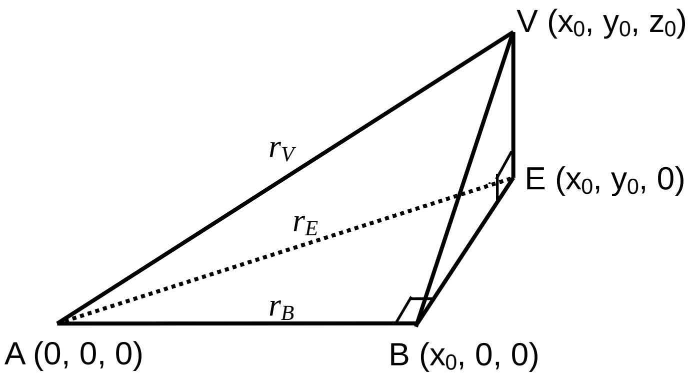

Figure S1 *ABEV* subsimplex with coordinate assignments for points *B, E,* and *V,* relative to atom *A.*

vertices in the cluster which completely encloses the cavity. Thus, the sum of volumes of Delaunay simplices corresponding to Voronoi vertices in a cavity yields the upper limit of the cavity volume. Then the cavity volume can be precisely calculated by subtracting the volume inside the exclusion zones of atoms from the total volume of a set of Delaunay simplices enclosing the cavity. In each Delaunay simplex, the simplex can be further divided into subsimplices with volume of $V_{ABEV} = S_V S_E \left| (\overrightarrow{AB} \times \overrightarrow{AE}) \cdot \overrightarrow{AV} \right|$.

Fig.S1 shows a subsimplex *ABEV,* in which r_V is the distance from the atom *A* to the Voronoi vertex *V*. r_E and r_B are the distances from *A* to the intersection of the Voronoi edge with the Delaunay face *E* and the midpoint of a Delaunay edge *B*, respectively.

$r_B = x_0$, $r_E = \sqrt{x_0^2 + y_0^2}$, $r_V = \sqrt{x_0^2 + y_0^2 + z_0^2}$

In order to calculate the cavity volume, the overlap volume V_c of the subsimplex with the exclusion sphere of radius r_c ($<r_V$) should be calculated and subtracted from the total V_t. Here the final expressions for the V_c calculation are given as follows:

1. When $r_c < x_0$, $V_c = \frac{r_c^3}{6}\left(2\theta - \pi/2 - \arcsin\left[\frac{(z_0^2 x_0^2 - y_0^2 r_V^2)}{r_E^2 (y_0^2 + z_0^2)}\right]\right)$.

2. When $x_0 < r_c < r_E$, $V_c = \frac{\theta}{2}\left(r_c^2 x_0 - \frac{x_0^3}{3}\right) - \frac{r_c^3}{6}\left(\frac{\pi}{2} + \arcsin\left[\frac{(z_0^2 x_0^2 - y_0^2 r_V^2)}{r_E^2 (y_0^2 + z_0^2)}\right]\right)$.

3. When $r_E < r_c < r_V$,

$$V_c = \frac{1}{2}\left(\theta - \frac{\pi}{2} + \arcsin\left[\frac{y_0}{\sqrt{r_c^2 - x_0^2}}\right]\right)\left(r_c^2 x_0 - \frac{x_0^3}{3}\right) + \frac{x_0 y_0}{6}\sqrt{r_c^2 - r_E^2}$$
$$+ \frac{r_c^3}{6}\arcsin\left[\frac{x_2^2 - y_2^2 - x_0^2}{r_c^2 - x_0^2}\right] - \frac{r_c^3}{6}\arcsin\left[\frac{(z_0^2 x_0^2 - y_0^2 r_V^2)}{r_E^2 (y_0^2 + z_0^2)}\right]$$

where $\theta = \arctan(z_0 / y_0)$, $x_2 = r_c x_0 / r_E$, and $y_2 = r_c y_0 / r_E$. This algorithm has been developed into an in-house program, and successfully used for the calculation of the cavity

volume in some metallic glasses[9].

3. Local atomic displacements

By using the method described above, we classify the atoms into two kinds: one is those in the "solid" regions and the other is those near the cavities. Therefore, we can further evaluate responses of atoms from two regions under the external stress. As shown in Fig.S2, The displacement is calculated using $\Delta r_i = \left| r_i(\varepsilon_{(k+1)\%}) - r(\varepsilon_{k\%}) \right|$, where k varies from 0 to 6 and $r_{\mathrm{i}}(k\%)$ is the atomic position of i atom at strain of k%. Note that the simulation box at strain of (k+1)% has been rescaled back to the same box size at k%, i.e., the elastic strain has been recovered when comparing the atomic displacement between two strains. Thus, the

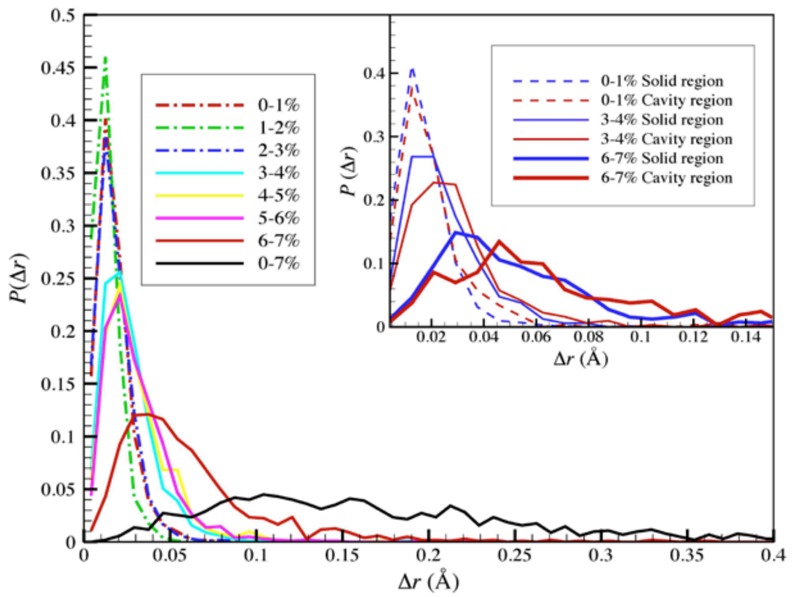

Figure S2 Displacement distribution function at different strains with respect to the case of 1% strain lower during loading, together with the one between 0% strain and 7% strain. The inset shows the difference in such displacements of atoms in the solid regions and cavity regions, respectively.

contribution to the displacement only comes from the inelastic part. Note that the displacements of atoms between every one percent of strain are very small when the overall strain is less than 3%, i.e., the average displacement is only 0.02 Å. With increasing tensile strain, the overall displacement increases as well as the maximum displacement. However, even considering the displacements of atoms between 0% stain and 7% strain, it is interesting to see that the majority displacement distributes below 0.4 Å, which strongly suggests that the atoms mainly exhibit a reversible elastic deformation and small inelastic displacements when the strain goes up to 7%. This also means that most of atoms still move inside the cages, only quite few can jump to the outside. The inset shows that the atoms in the cavity regions can move easily compared to those in the solid regions, thereby accumulating relatively large atomic displacements.

4. Voronoi polyhedra around Nb atoms

Using a cutoff of 3.6 Å in Voronoi tessellation method, the local responses of polyhedra centered by Nb atoms are also estimated. Fig. S3 illustrates the fraction changes of Voronoi polyhdra centered by Nb atoms with increasing the total strain. Fig. S4 (a) and (b) show the major polyhedra centered by Nb atoms are <0,1,10,4> and <0,2,8,4>. Another two kinds of polyhedra <0,1,10,3> and <0,0,12,3> also exhibit rather large changes in amount of fractional changes. A general evolution trend for the Nb-centered polyhedra under tension seems to go in this way, i.e., <0,1,10,4> and <0,1,10,3> evolve to <0,0,12,3> and <0,2,8,4>. When tensile strain is larger than 5 %, the fraction of polyhedron <0,1,10,4> in the solid regions decreases with increase of polyhedra <0,2,8,4> in the cavity regions significantly, as the phenomenon

observed in liquid melts, which could make excess free volume migrate from inside clusters to the outside, promoting the formation of cavities.

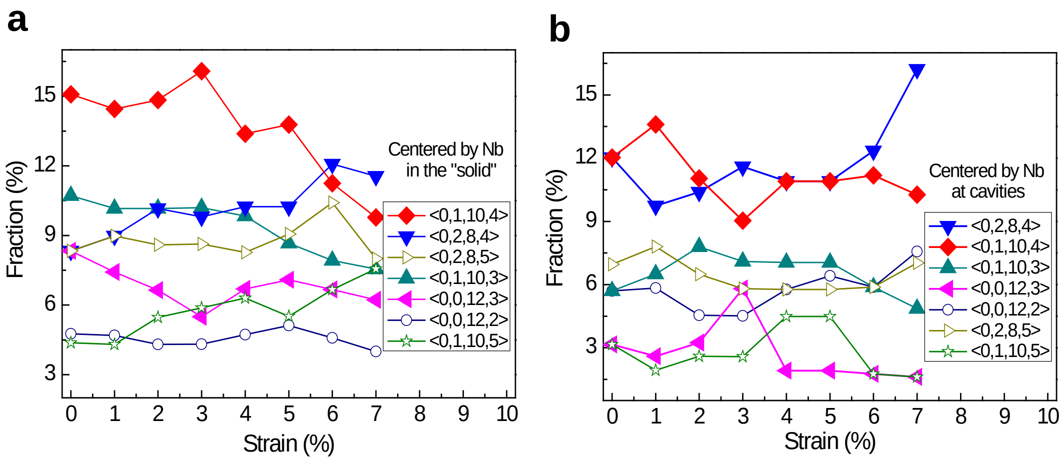

Figure S3 Fractions of Voronoi polyhedra centered by Nb atoms (a) in the "solid" regions and (b) near the cavities changing with tensile strain, respectively.

5. Changes in bond order parameters

As suggested by Steinhardt *et al.* [10], local bond orientational order parameter q_{lm}, calculated by summing up the spherical harmonics of all atomic bonds between the central atom and its nearest neighbors, is useful for determining the local orientation order of atoms. $q_{lm}(i) = \frac{1}{N_b(i)} \sum_{j=1}^{N_b(i)} Y_{lm}\left(\vec{r}_{ij}\right)$, where $N_b(i)$ is the number of the nearest neighbors of the atom, r_{ij} is the vector from atom i to atom j, and $Y_{lm}(\vec{r}_{ij})$ are spherical harmonics. More recently, a modified average version about the vector $\overline{q}_{lm}$ has been proposed [11], which has improved accuracy to identify the local ordering. $\overline{q}_{lm}(i) = \frac{1}{\tilde{N}_b(i)} \sum_{k=0}^{\tilde{N}_b(i)} q_{lm}(k)$, where $\tilde{N}_b(i)$ includes all neighbors of atom i, and $k = 0$ denotes the atom i itself. Thus, the average form of the local bond order parameters can be expressed as: $\overline{q}_l(i) = \sqrt{\frac{4\pi}{2l+1} \sum_{m=-l}^{l} \left|\overline{q}_{lm}(i)\right|^2}$.

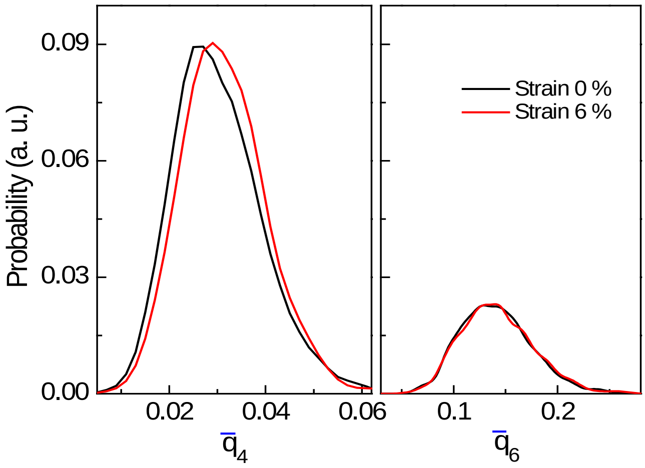

Figure S4 Comparison in average local bond order parameters $\overline{q}_4$ and $\overline{q}_6$ between zero strain and 6% strain.

One can see that the $\overline{q}_4$ value slightly shifts to the large values with strain is up to 6%, indicating the increased local ordering induced by tensile stress. Since the $\overline{q}_6$ parameter is more sensitive to the contribution coming from the icosahedron-like clusters that have rather high rigidity against deformation, the $\overline{q}_6$ parameter exhibits little changes between zero and 6% strain here, good agreement with the results in Fig. 5(c) in the main text.

6. Elastic behavior of $Cu_{50}Zr_{50}$ metallic glass reproduced by classic MD

We tested the classic Ni-Nb potentials[12] reported in the literature and found that they are not accurate to simulate the mechanical properties of Ni-Nb MGs. In contrast, Cu-Zr alloys have been intensively studied by classic MD since the well developed Cu-Zr potentials are more reliable. Thus, we also adopted the classic MD by LAMMPS code [13] and widely used Cu-Zr potential[14] to simulate the elastic behavior of a $Cu_{50}Zr_{50}$ MG. The sample size of (x) 8.5 nm*(y) 17 nm*(z) 5.8 nm, containing 46679 atoms, was first equilibrated at 2000 K for 2

ns and then cooled down to 50 K at a cooling rate of 10^{11} K/s with 3D periodic boundary conditions (PBCs), NPT ensemble and Nose-Hoover thermostat. In uniaxial tension and compression, PBCs were also imposed and a constant strain rate 10^8/s was used to draw/compress the atoms along the y direction at T = 50 K. Fig. S5(a) shows the stress-strain curves produced by the classic MD. One can see that the elastic strain limit under tension is larger than that under compression. Also, when the stress is over 0.8 GPa, the strain under tension becomes more deviated from the linear relationship than in compression, indicating that the tension more favors the viscoelasticity of MGs. Fig. S5(b) illustrates the total volume change during tension and compression. Note that tension increases the cell volume while compression decreases it. The volume change of the subatomic cavities almost follows the same way with the total volume change. However, some cavitation[15] may take place when the strain is over 3%, showing a rapid increase of cavity volume in Fig.S5(c). Fig. S6 gives the von Mises strain distribution during tension and compression when the strain increases up to 6%, showing more atoms have large von Mises strain under compression than tension when the strain is over 5%. The similar variation trend was kept when each configuration was relaxed 2 ns at 50 K with NVT ensemble. A broad von Mises strain distribution indicates that the atoms already exhibit heterogeneous response to the external stress even in the pure elastic region, showing the major feature of non-affine deformation. Thus, from this point of view, the classic MD is not precise as the *ab initio* DFT to capture the main feature of pure elastic deformation of MGs.

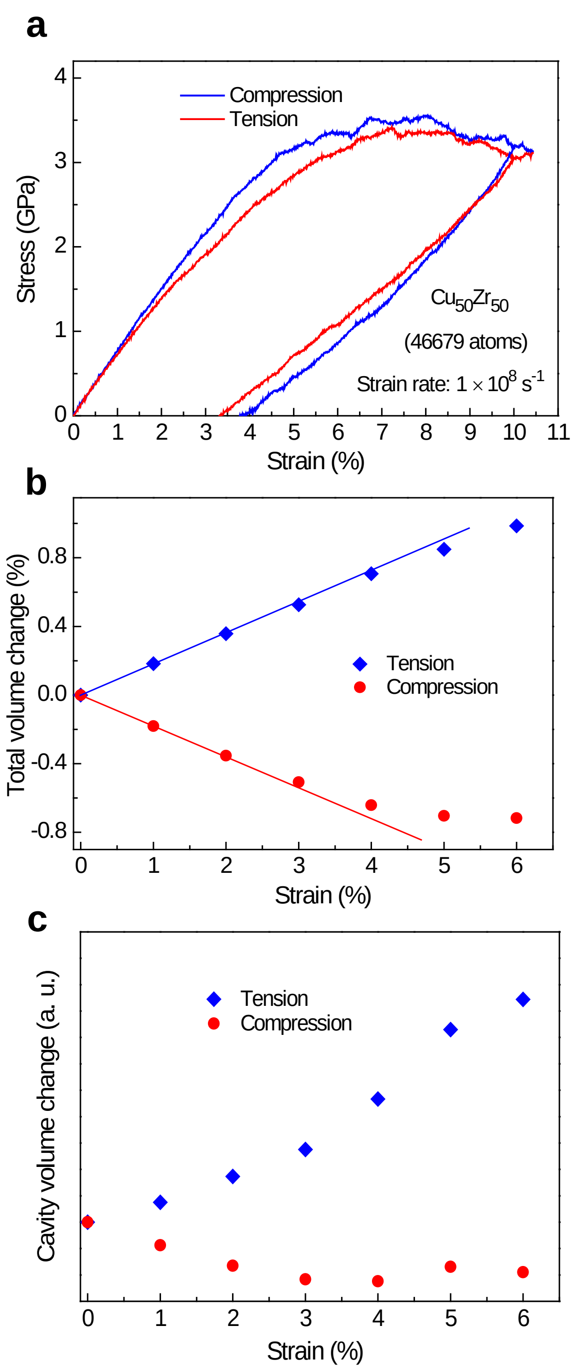

Figure S5 (a) Stress-strain curves of the $Cu_{50}Zr_{50}$ MG upon tension and compression produced by the classic MD with a strain rate of 10^8/s. and (b) Total volume change and (c) Cavity volume change with tensile or compressive strains.

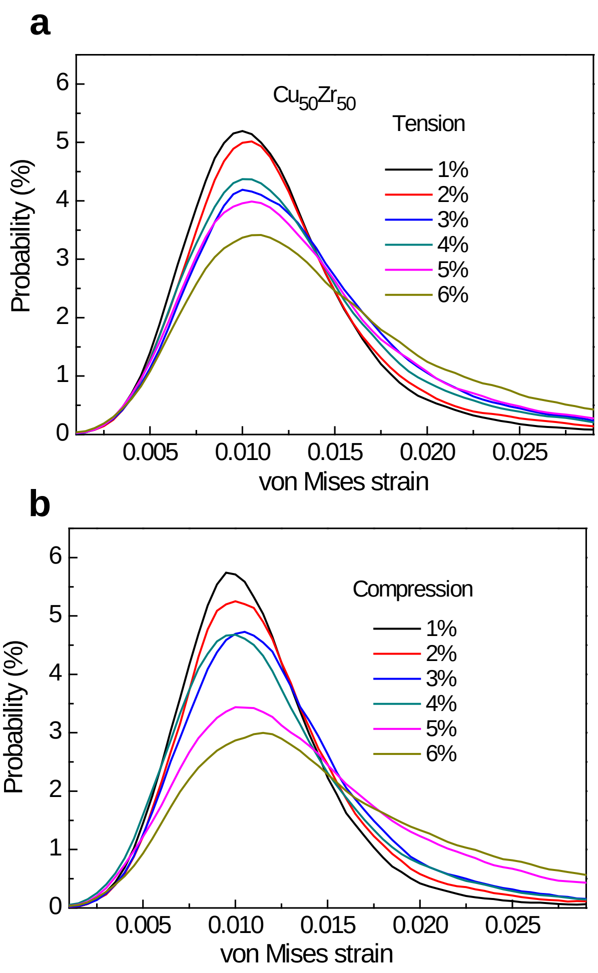

Figure S6 (a) Distribution of von Mises strain changing with tensile strain (a) and compressive strain for a $Cu_{50}Zr_{50}$ MG. It shows that the von Mises strain obtained by the classic MD distributes in a broad range, even the strain is less than 2%. When the strain is over 5%, more atoms have larger von Mises strain in compression than in tension.

References:

[1] G. Kresse and J. Furthmuller, Phys. Rev. B **54**, 11169 (1996).

[2] P.E. Blöchl, Phys. Rev. B **50**, 17953 (1994).

[3] Y. Wang and J.P. Perdew, Phys. Rev. B **44**, 13298 (1991).

[4] J. P. Perdew, K. Burke, and M. Ernzerhof, Phy. Rev. Lett. **77**, 3865 (1996).

[5] A. P. Hammersley, S. O. Svensson, M. Hanfland, A. N. Fitch, and D. Häusermann, High Press. Res. **14**, 235(1996).

[6] I.-K. Jeong, J. Thompson, A. M. P. Turner, and S. J. L. Billinge, J. Appl. Crystallogr. **34**, 536 (2001).

[7] M.H. Cohen and D. Turnbull, J. Chem. Phys. **31**, 1164 (1959).

[8] S. Sastry, D.S. Corti, and P.G. Debenedetti, *et al*., Phys. Rev. E **56**, 5524 (1997).

[9] H.W. Sheng, E. Ma and M. Kramer, JOM, **64**, 586 (2012).

[10] P. J. Steinhardt, D. R. Nelson, and M. Ronchetti, Phys. Rev. B **28**, 784 (1983).

[11] W. Lechner and C. Dellago, J. Chem. Phys. **129**, 114707 (2008).

[12] K.P. Tai, L.T. Wang, B.X. Liu, J. Appl. Phys. **102**, 124902 (2007).

[13] S. Plimpton, J. Comput. Phys. **117**,1 (1995).

[14] M. I. Mendelev, D. J. Sordelet, and M. J. Kramer, J. Appl. Phys. **102**, 043501 (2007).

[15] P. Guan, S. Lu, M.J.B. Spector, P. K. Valavala, M.L. Falk, Phys. Rev. Lett. **110**, 185502 (2013).
